# Relationship between self-reported hearing and vision problems, cognitive decline, depressive symptoms, and life satisfaction in older adults: a retrospective observational study

**DOI:** 10.1186/s12889-024-18624-5

**Published:** 2024-04-23

**Authors:** Yuan Chen

**Affiliations:** Department of Special Education and Counselling, The Education University of Hong Kong, Tai Po, New Territories, Hong Kong SAR, China

**Keywords:** Older adults, Cognition, depressive symptoms, Life satisfaction, Hearing impairment, Vision impairment

## Abstract

**Background:**

Sensory impairment in older adults is associated with cognitive decline, elevated depressive symptoms, and low levels of life satisfaction. However, these relationships are usually investigated separately and in pairs. This study examined these relationships comprehensively, for the first time.

**Methods:**

The analysis included 5,658 community-dwelling older adults from the China Health and Retirement Longitudinal Study (aged 50 to 108 years, 52.1% male) who completed the Jorm Informant Questionnaire Cognitive Decline in the Elderly and the Center for Epidemiological Studies-Depression-short form. A questionnaire was used to collect information on hearing, visual status, and life satisfaction. Structural equation modelling was used to examine the direct and indirect relationships between these variables.

**Results:**

Self-reported hearing and vision problems are directly associated with cognitive decline and elevated depressive symptoms. In addition, hearing and vision problems are indirectly related to cognitive decline through elevated depressive symptoms. Although hearing and vision problems had no direct effect on life satisfaction, they were indirectly associated with life satisfaction through cognitive decline and depressive symptoms.

**Conclusions:**

This study provides the first epidemiological evidence of the comprehensive relationships between hearing and vision problems, cognitive decline, depressive symptoms, and life satisfaction. When older adults report hearing and/or vision problems, clinicians and caregivers should be aware of the concurrence of declined cognition, elevated depressive symptoms, and compensated life satisfaction. Future studies should examine the causal relationships and potential mechanisms of these relationships.

## Background

According to World Health Organization (WHO) data, the proportion of older adults (≥ 60 years of age) will nearly double from 12% in 2015 to 22% in 2050. This rapid growth in the population of older adults has profound implications on public health, requiring a remarkable demand for health care delivery for age-related services.

Globally, hearing loss is a significant concern, affecting around 466 million people, including 432 million adults, most of whom are in low- and middle-income countries [1]. Concurrently, vision impairment impacts over 1 billion people worldwide, primarily due to unaddressed refractive errors and cataracts, with this number anticipated to rise with an aging population [2]. The high prevalence of these sensory impairments highlights the necessity for comprehensive health strategies tailored for older adults.

Prior research has demonstrated significant associations between vision and hearing impairments and an increased risk of dementia, cognitive decline [3, 4], depressive symptoms [5–7], and reduced life satisfaction [8, 9] among older adults. Moreover, older adults exhibiting depressive symptoms present an increased risk of cognitive impairment [10, 11]. Life satisfaction has also been linked with cognitive decline [12] and depression [13]. However, these relationships have been examined in isolation. The comprehensive interplay between sensory decline, cognition, depressive symptoms, and life satisfaction has yet to be fully explored, leaving the direct and indirect effects of these relationships largely unclear.

The Biopsychosocial Model [14] proposes that biological, psychological, and social factors all significantly contribute to human functioning in the context of disease or illness. It suggests that vision and hearing problems (biological factors) may directly lead to elevated depressive symptoms and may also impact cognition (psychological factors). These issues could subsequently result in decreased life satisfaction, particularly if they lead to social isolation or a reduced ability to participate in previously enjoyable activities (social factors).

**Fig. 1:**
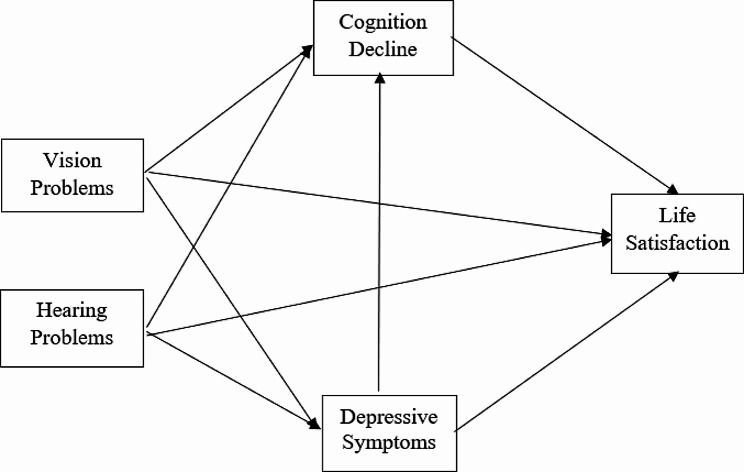
A hypothesized model of relationships among hearing and vision problems, cognitive decline, depressive symptoms, and life satisfaction

After considering these factors and existing research, we proposed the following conceptual model illustrated in Fig. 1. Our hypotheses were as follows: (1) self-reported hearing and vision impairments would demonstrate significant associations with cognitive decline, elevated depressive symptoms, and reduced life satisfaction; (2) hearing and vision problems would have an indirect relationship with cognitive decline through their impact on depressive symptoms; and (3) cognitive decline and depressive symptoms would indirectly affect life satisfaction through cognitive decline and depressive symptoms.

## Methods

### Study participants

The participants were from the China Health and Retirement Longitudinal Study (CHARLS) [15]. It established a nationally representative sample of residents in mainland China aged above 45 years and consisted of four waves of data collection: Wave 1 in 2011, Wave 2 in 2013, Wave 3 in 2015, and Wave 4 in 2018. The CHARLS research spanned 28 provinces and incorporated 150 counties or districts along with 450 villages or urban communities. The researchers employed a multistage stratified probability-proportionate-to-size sampling method. The sampling was stratified by region, and within each region, further stratification was done by urban districts or rural counties and per capita statistics on Gross Domestic Product (GDP) (for a detailed explanation of this method, refer to Zhao et al. [16]). In each region, the CHARLS researchers gathered comprehensive lists of all residences in each region and used a random selection process to choose which households would participate in the survey. Only data from Wave 4, which contains full sets of variables (sensory problems, depressive symptoms, cognitive decline, and life satisfaction), were included for analysis in the current study. The CHARLS wave 4 originally included 19,816 individuals from households including at least one person aged 45 years or older. After excluding participants aged < 50 years and those who did not report sensory status, cognitive, depressive, and life satisfaction, a total of 5,658 participants were included in the analysis. We selected participants aged 50 and above due to the current retirement policy in China. According to this policy, men typically retire at 60, while the retirement age for women is 55 for white-collar workers and 50 for blue-collar workers [17]. Ethical approval for all CHARLS waves was granted by the institutional review board (IRB) at Peking University. The IRB approval number for the main household survey, including anthropometrics, was IRB00001052-11015 and the IRB approval number for biomarker collection was IRB00001052-11014. Written consent was obtained before data collection.

### Study assessments

#### Self-reported hearing and vision problems

Self-reported sensory problems were evaluated using two questions: (1) Do you have hearing problems (yes/no)? (2) Do you have vision problems (yes/no)?

#### Cognitive decline

The Informant Questionnaire on Cognitive Decline in the Elderly (IQCODE) [18] played a crucial role in our study, enabling the assessment of cognitive decline in older adults via a detailed 26-item questionnaire. This tool uniquely evaluates changes in everyday cognitive functions, utilizing a 5-Point Likert scale that ranges from 1 (“much better”) to 5 (“much worse”) for scoring, with higher scores indicating more significant cognitive decline. The IQCODE has been adapted and standardized for the Chinese population [19].

A defining characteristic of the IQCODE is its dependence on informant reports rather than direct participation from the older adults themselves. While reliance on informant reports may not always yield a comprehensive or entirely objective evaluation of cognitive decline—given it is contingent on the informants’ perceptions and the quality of their relationship with the assessed participants—the IQCODE circumvents the need for direct participation from older adults. Therefore, the results are not affected by the sensory capabilities of the elderly during the test administration [19]. The IQCODE has been shown to slightly outperform the Mini-Mental State Examination (MMSE) as a screening tool for dementia [19].

#### Depressive symptoms

The Center for Epidemiological Studies-Depression (CES-D) short form is a concise 10-item survey designed to assess the frequency of depression-related symptoms experienced over the past week [20]. Symptoms include restless sleep, feelings of hopelessness, and problems with concentration. Each item is rated on a scale of 1–4, where 1 signifies rare or no occurrence, 2 indicates some or a little occurrence, 3 represents occasional or moderate occurrence, and 4 denotes most or all the time. A higher score implies more severe depressive symptoms. The CES-D short form has been standardized for use with older Chinese individuals [21]. In our current analysis, we summed the responses and treated them as continuous data.

#### Life satisfaction

Life satisfaction was assessed utilizing a single-item measure, a method that has been widely employed in sociological and psychological research [22–24]. Participants were asked to evaluate their overall satisfaction with life by responding to the question: “Please think about your life as a whole. How satisfied are you with it?” The responses were recorded using a 5-point Likert scale, where 1 corresponded to being ‘completely satisfied,’ 2 was ‘very satisfied,’ 3 was ‘somewhat satisfied,’ 4 was ‘not very satisfied,’ and 5 indicated being ‘not at all satisfied.’ This measure offered a comprehensive overview of an individual’s subjective assessment of their life satisfaction [23] a critical marker of psychological well-being [22]. In this study, a higher score on this scale denoted lower life satisfaction.

#### Covariates

A questionnaire was used to collect demographical and health-related information. This included age (treated as a continuous variable), sex (male/female), and the presence or absence of certain health conditions (hypertension, dyslipidemia, diabetes, and stroke), along with smoking (smokers/non-smokers) and drinking status (drinkers/non-drinkers). Marital status was categorized into two groups: 1. Married and 2. Single. Education level was divided into four categories: 1. No formal education (illiterate), 2. Up to Primary School, 3. Up to Junior Middle School, 4. Senior Middle School or beyond. These categories were then redefined into three dummy variables, using the ‘illiterate’ category as the reference. Given the disparities in healthcare access between rural and urban areas, we also incorporated the variable of region (urban/rural) into our analysis (see Table 1).

### Data analysis

Descriptive statistics were used to characterize the demographics, cognitive decline, depressive symptoms, and life satisfaction. Independent-sample t-tests were used to examine whether there were differences in demographics, cognitive decline, depressive symptoms, and life satisfaction between those with and without hearing/vision problems. Pearson’s correlation coefficients were calculated to examine the relationships among cognitive decline, depressive symptoms, and life satisfaction.

Structural equation modelling (SEM) was then conducted to test the hypothesized direct and indirect relationships between sensory disabilities, cognitive decline, depressive symptoms, and life satisfaction after controlling for demographics (i.e., age, sex, education level, marital status, Hypertension, dyslipidemia, diabetes, stroke, region, drinking and smoking status) (see Fig. 1). SEM, unlike traditional regression models, offers a more comprehensive understanding of complex relationships among multiple variables by allowing for the simultaneous examination of direct and indirect effects [25, 26]. This is particularly beneficial in this study as it allows us to understand not only the direct impact of sensory disabilities on cognitive decline, depressive symptoms, and life satisfaction but also their indirect effects via other variables [25].

The maximum likelihood estimation method was used to examine model parameters. The comparative fit index (CFI), Tucker-Lewis index (TLI), the root mean square error of approximation (RMSEA), and the standardized root-mean-square residual (SRMR) were used to examine the model fit. A good model fit is suggested by CFI and TLI values of 0.95 or greater, an RMSEA value of 0.05 or lower, and an SRMR value of 0.08 or lower [27].

To examine indirect effects, a bootstrapping analysis using 1,000 bootstrap samples was conducted. Bias-corrected 95% confidence intervals (CIs) were used to determine the significance of indirect effects, which were considered significant if the 95% CI did not cross zero. All analyses were performed using SPSS version 26 and Amos (version 26). The constructs of cognitive decline, depressive symptoms, and life satisfaction in this study were all directly measured variables rather than latent ones, assessed through standardized and validated tests that yielded quantifiable results for each construct.

## Results

### Participant demographics

The mean age was 67.54 years (± 6.00), ranging from 50 to 108 years. 52.1% (*n* = 2,947) of participants were male. A total of 4.2% (*n* = 235) and 7.2% (*n* = 405) respondents reported vision and hearing problems, respectively. The majority of the participants had an education level below senior middle school, accounting for 87.7%, and were married, representing 84.7% of the sample. Participants who reported hearing and/or vision problems were typically older, resided in rural areas, and suffered from dyslipidemia and diabetes. Additionally, these individuals were more likely to be smokers as compared to those who did not have any hearing or vision problems (Table 1).

**Table 1:** Characteristics of participants

Variables	*N*	Frequency	*t* value for difference between groups with and without hearing problems	t value for difference between groups with and without vision problems
**Gender**			1.12	3.28**
Male	2,947	52.1%		
Female	2,712	47.9%		
**Education level**				
1. No formal education (illiterate) (reference category)	1344	23.8%		
2. ≤ Primary School	2563	45.3%	-0.15	0.07
3. ≤Junior middle school	1057	18.7%	-2.74**	-3.75***
4. ≥Senior middle school	694	12.3%	-1.29	-3.00**
**Marital status**			1.26	2.46*
1. Married	4795	84.7%		
2. Single	863	15.3%		
**Region**			3.07**	0.63
Urban	1743	30.8		
Rural	3915	69.2		
**Hypertension**			-1.03	-2.45*
Yes	673	11.9%		
No	4985	88.1%		
**Dyslipidemia**			-3.09**	-2.44*
Yes	592	10.5%		
No	5066	89.5%		
**Diabetes**			-2.33*	-2.40*
Yes	337	6.0%		
No	5321	94.0%		
**Stroke**			0.95	-1.42
Yes	339	6.0%		
No	5319	94%		
**Smoking Status**			0.96	-2.24*
Smoker	1590	28.1%		
Non-smoker	4068	71.9%		
**Drinking Status**	1971	34.8%	0.66	2.66**
Drinkers	3687	65.2%		
Non-drinkers				

*p < 0.05, **p < 0.01, *** *p* < 0.001

The IQCODE scores varied from 28 to 130, with a mean of 87.38 and a standard deviation of 13.24. The CES-D scores had a mean of 19.37 and a standard deviation of 5.38, while the life satisfaction scores averaged at 2.68 with a standard deviation of 0.76. The skewness and kurtosis for the IQCODE, CES-D, and life satisfaction scores were all below 1.3, adhering to the acceptable ranges proposed by Weston and Gore [28] - that is, skewness should not exceed 3.0, and kurtosis should not exceed 10.0. These values suggest that the data distribution closely approximates a normal distribution. Independent t-tests showed that those who reported vision problems showed significantly greater cognitive decline (*t* = 6.81, *p* < 0.001), higher levels of depressive symptoms (*t* = 6.38, *p* < 0.001), and lower levels of life satisfaction (*t* = 3.20, *p* = 0.002) compared to those without vision problems. Similarly. those who reported hearing problems exhibited greater cognitive decline (*t* = 7.37, *p* < 0.001), higher levels of depressive symptoms (*t* = 4.42, *p* < 0.001), and lower levels of life satisfaction (*t* = 2.64, *p* = 0.008). Additionally, higher life satisfaction was significantly correlated with lesser cognitive decline (*r* = 0.08, *p* < 0.001) and lower levels of depressive symptoms (*r* = 0.23, *p* < 0.001). Higher levels of depressive symptoms were also significantly associated with greater cognitive decline (*r* = 0.16, *p* < 0.001).

### Direct effects

**Fig. 2:**
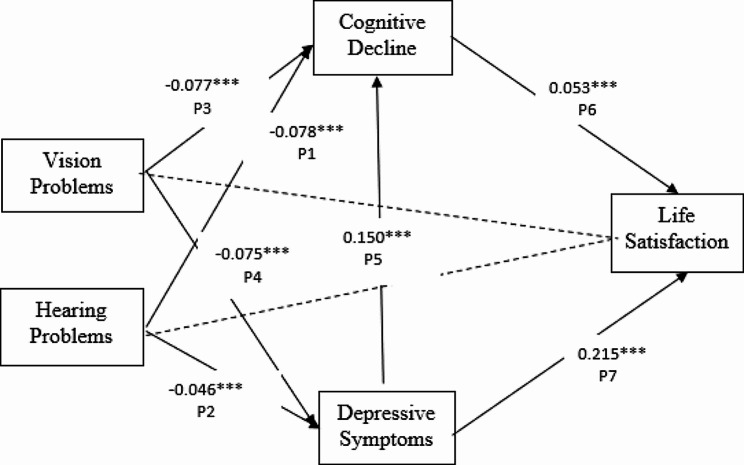
Illustrates the standardized path coefficients of the proposed model. The solid lines denote significant paths, while the dashed lines indicate paths that are not significant. Each path is labeled with “Pn”, where “n” represents the path number. For example, P1 refers to the path that leads from hearing problems to cognitive decline

SEM (see Fig. 2) showed a good model fit: χ^2^ = 291.024 (*df* = 76, *p* < 0.001), TLI = 0.954, CFI = 0.977, RMSEA = 0.022., and SRMR = 0.021. Table 2 presents the direct effects of the hypothesized model. Older adults who reported hearing problems showed more severe cognitive decline (*β* = -0.078, *p* < 0.001) and elevated depressive symptoms (*β*=-0.046, *p* < 0.001) compared to those without reported hearing problems. Similarly, those who reported vision problems exhibited more severe cognitive decline (*β*=-0.077, *p* < 0.001) and elevated depressive symptoms (*β*=-0.075, *p* < 0.001) compared to those who did not report vision problems. However, neither hearing (*β* =-0.015, *p* = 0.260) nor vision problems (*β* = -0.016, *p* = 0.214) were significantly associated with lower life satisfaction. In addition, cognitive decline was significantly associated with elevated depressive symptoms (*β* = 0.150, *p* < 0.001) and lower levels of life satisfaction (*β*=-0.053, *p* < 0.001). Depressive symptoms and life satisfaction were also positively correlated (*β* = 0.215, *p* < 0.001).

### Indirect effects

Table 3 shows the indirect effects of the hypothesized model. Bootstrapping analysis indicated that the effects of vision problems and hearing problems on cognitive decline were mediated by depressive symptoms. However, vision problems and hearing problems were not directly related to life satisfaction. Sensory problems can significantly affect life satisfaction indirectly in three ways: (1) via its effects on cognitive decline, (2) via its effects on depressive symptoms, (3) and via its effects on cognitive and depressive symptoms. In addition, a significant indirect effect of depressive symptoms on life satisfaction via cognitive decline was also observed.

**Table 2:** Unstandardized and standardized path coefficients for the path analysis-direct effects

	Unstandardized B (SE)	Standardized β
P1: Hearing problems → Cognitive decline	-4.017***	-0.078***
P2: Hearing problems → Depressive symptoms	-0.955***	-0.046***
P3: Vision problems → Cognitive decline	-5.119***	-0.077***
P4: Vision problems → Depressive symptoms	-2.107***	-0.078***
P5: Depressive symptoms → Cognitive decline	0.370***	0.150***
P6: Cognitive decline → Life satisfaction	0.003***	0.053***
P7: Depressive symptoms → Life satisfaction	0.030***	0.215***
P8: Hearing problems → Life satisfaction	-0.044	-0.015
P9: Vision problems → Life satisfaction	-0.062	-0.016

*p < 0.05, **p < 0.01, *** *p* < 0.001

**Table 3:** Unstandardized and standardized path coefficients for the path analysis-indirect effects

	Unstandardized B (SE)	95% confidence interval
Vision problems→ Depressive symptoms → Cognitive decline P4*P5	-0.780***	-1.151 - -0.457
Vision problems→ Cognitive decline → Life satisfaction P3*P6	-0.016***	-0.027 - -0.007
Vision problems→ Depressive symptoms → Life satisfaction P4*P7	-0.064***	-0.091 - -0.039
Vision problems→ Depressive symptoms → Cognitive decline → life satisfaction P4*P5*P6	-0.002***	-0.004 - -0.001
Hearing problems → Depressive symptoms → Cognitive decline P2*P5	-0.353***	-0.617 - -0.150
Hearing problems → Cognitive decline → life satisfaction P1*P6	-0.012***	-0.022 - -0.006
Hearing problems → Depressive symptoms → life satisfaction P2*P7	-0.029***	-0.048 - -0.012
Hearing problems → Depressive symptoms → Cognitive decline → life satisfaction P2*P5*P6	-0.001***	-0.002–0.000

*** *p* ≤ 0.001

## Discussion

### Sensory decline and cognitive decline

This study adds to the growing literature linking poor hearing [29–32] and vision [3, 33, 34] to cognitive decline. However, the causal mechanisms underlying the association between sensory and cognitive decline remain unclear. Wayne and Johnsrudue [35] summarized four potential hypotheses:1) cognitive load on perception hypothesis, which implies that cognitive decline causes sensory decline. 2) the information-degradation hypothesis, suggesting that auditory sensory decline causes impoverished (but reversible) cognitive function; 3) the sensory deprivation hypothesis, indicating that auditory sensory impairment leads to more permanent cognitive decline; and 4) the common cause hypothesis, proposing that a third variable (e.g., cerebrovascular disease; social relations, and genetics) underlines cognitive decline and hearing (and other sensory modalities) [35, 36]. As both auditory and visual senses were found to be significantly related to cognitive decline, the findings seem to support the common cause hypothesis. However, the current study could not determine the third variable. In addition, the other hypothesis cannot be rejected, considering the cross-sectional nature of the study.

### Sensory decline and depressive symptoms

We found that older adults with hearing or vision problems were more likely to exhibit elevated depressive symptoms, which was also reported in a previous study [6, 9, 37, 38]. Sensory decline can cause dramatic changes in people’s lives, especially in daily life activities, such as travelling outside and communicating with others. Depression may develop when older adults struggle to deal with the consequences of such declines [9]. In addition, changes in activities of daily living can further affect social interaction which is an indispensable part of life. Degraded social interactions can make life less pleasurable and less meaningful, resulting in loneliness and depression [39]. Rovner et al. [40] found that the association between vision problems and depressive symptoms could be attenuated by using an integrated vision rehabilitation and mental health program, suggesting that this association could be modifiable [40]. Therefore, clinicians should be aware of this association and provide referrals to address the mental health needs of older adults with self-reported hearing and vision problems when appropriate [6].

In addition, this study found that hearing and vision problems were indirectly related to cognition through elevated depressive symptoms. Previous studies have reported a significant relationship between cognition and depression. For example, a systemic review conducted by Rock et al. (2014) demonstrated that cognitive deficits were common in patients with depression [41]. A meta-analysis found that depression was associated with an increased risk of subsequent dementia [42]. Our study further substantiates the existing understanding of the mediating role of depressive symptoms in the relationship between cognitive decline and sensory impairment [35]. Wayne and Johnsrude [35], for instance, proposed elevated depressive symptoms as a potential mediator in the sensory impairment deprivation hypothesis.

### Sensory decline and life satisfaction

Our study failed to find a direct effect of the sensory decline on life satisfaction. Instead, hearing and vision problems had indirect effects on life satisfaction via their effects on cognitive decline and depressive symptoms. Bourque et al. [43] examined the relationship between self-reported sensory decline and life satisfaction in 826 older French-speaking participants and found that self-reported sensory decline contributed significantly to life satisfaction [43]. However, depression was not included in the analysis in Bourque et al. [43], which may explain the difference in the findings between the two studies. Previous studies have found significant associations between sensory decline and life satisfaction [8, 9], life satisfaction and cognitive decline [12], and life satisfaction and depression [12]. We found that cognition and depressive symptoms might mediate the relationship between sensory decline and life satisfaction. This suggests that life satisfaction in older adults with sensory decline can be improved by interventions to alleviate cognitive decline and depressive symptoms.

### Limitations

This study has several limitations. First, vision and hearing functions were evaluated based on self-reported items without a detailed assessment of visual and auditory acuity. Although self-reported vision and hearing status are prone to recall and social desirability biases [6], they are easy to measure and have been widely used in population-based studies. In addition, self-reported vision and hearing problems may be better indicators of cognitive decline, depressive symptoms, and low levels of life satisfaction than visual and auditory acuity. For example, Zheng et al. [44] found that self-reported vision problems were significantly associated with elevated depressive symptoms compared with visual acuity. Similarly, Hickson et al. [45] suggested that self-reported hearing problems could offer a more sensitive gauge of hearing-related functional impairments in older adults’ daily lives compared to audiometric hearing assessment. These studies imply that self-reported hearing and vision problems may represent a unique and valuable construct for identifying individuals at risk of cognitive decline and high levels of depressive symptoms.

Second, only 7.2% and 4.2% of the participants reported hearing and vision problems, respectively. These percentages were lower than the prevalence of hearing and visual impairment measured using standard examinations in China. To illustrate, data from the Second National Sample Survey on Disability conducted in 2006 revealed that around 11% of individuals aged 60 and above suffered from moderate to severe hearing disabilities [46]. Additionally, using the World Health Organization’s best-corrected visual acuity criteria, about 6.1% of people aged 45 and older demonstrated symptoms of low vision or blindness within China [47, 48]. Low prevalence of self-report vision and hearing problems may be attributed to several cultural and social reasons: Sensory decline may be seen by Chinese older adults as being a part of their elderly character [29]; therefore, many Chinese older adults with sensory decline may not consider them as a “problem”. In addition, older Chinese individuals usually live with their families and are accorded respect, which means that their family members typically alter their communication behavior (e.g., speaking louder, closer, slower, and repetitively) to ensure mutual understanding [49]. These practices prevent sensory decline from becoming a “problem”. Therefore, self-reported sensory decline may reflect not only sensory acuity but also self-rating as to whether it has severely affected daily life [50].

Third, the cross-sectional nature of the study prevented the examination of the causal relationships among these factors. This is because the first three waves of the data did not examine depressive symptoms or cognitive decline. We are waiting for wave 5 data to conduct a longitudinal analysis to further understand the causal and temporal relationships among self-reported sensory problems, cognitive decline, depressive symptoms, and life satisfaction in older Chinese people. Nevertheless, the preliminary results reported here emphasize the importance of self-reported sensory problems in cognitive decline, depressive symptoms, and life satisfaction.

## Conclusions

The study suggested that those who reported hearing and/or vision problems were more likely to exhibit lower cognitive decline and elevated depressive symptoms. In addition, self-reported hearing and vision problems were indirectly related to cognitive decline through elevated depressive symptoms. Although hearing and vision problems are not directly associated with life satisfaction, they are indirectly related to life satisfaction through cognitive decline and depressive symptoms. Therefore, clinicians facing older adults with reported hearing and/or vision problems should be aware of a concurrent decline in cognition, elevated depressive symptoms, and compensated life satisfaction. Preventive interventions and treatments for cognitive decline and elevated depressive symptoms should be considered to improve life satisfaction in this population.

## Data Availability

The data sets analysed in this paper are available publicly at the website https://charls.pku.edu.cn/en/.
